# New Frontiers in modeling the lipedema microenvironment *in vitro*

**DOI:** 10.3389/fcell.2026.1816014

**Published:** 2026-04-21

**Authors:** Khushi Soni, Rosalyn D. Abbott

**Affiliations:** Department of Biomedical Engineering, Carnegie Mellon University, Pittsburgh, PA, United States

**Keywords:** 3D cell culture, adipose tissue engineering, in vitro modeling, lipedema, pathogenesis

## Abstract

Lipedema is a chronic and often debilitating adipose tissue disorder that primarily affects women. The disease is characterized by disproportionate and symmetrical accumulation of subcutaneous fat in the extremities. Despite the high prevalence of lipedema, which affects ∼10% of women, and its significant impact on patient quality of life, lipedema is understudied and often misdiagnosed as other disorders (obesity or lymphedema). In this review, we explore the current understanding of lipedema through clinical, tissue, and cellular lenses, and examine suspected pathological mechanisms, including hormonal influences (such as estrogen), adipocyte hypertrophy and hyperplasia, increased extracellular matrix (ECM) fibrosis, and specialized immune cell involvement, including M2 macrophage infiltration. Recent advancements in adipose tissue engineering, including organoids, fat-on-a-chip platforms, and the use of induced pluripotent stem cells (iPSCs) are explored as platforms to study lipedema pathogenesis.

## 1 Introduction

**FIGURE 1:**
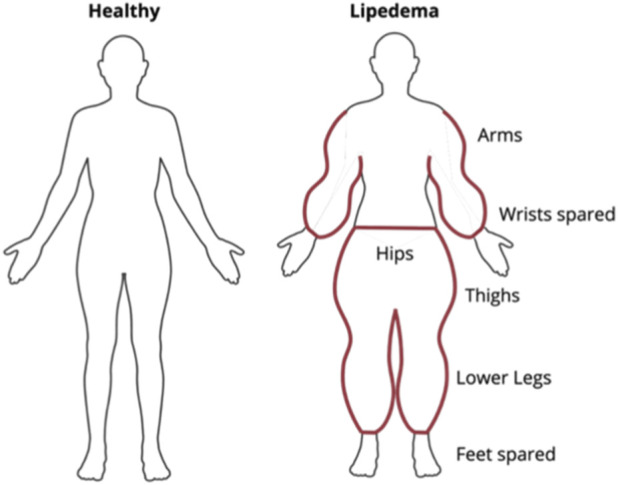
Lipedema is characterized by disproportionate, symmetrical accumulation of fatty tissue in the lower extremities, particularly the hips, thighs, and calves. Approximately 80% of affected women also exhibit fat accumulation in the upper arms. Notably, the hands and feet are typically spared. This abrupt transition at the wrists and ankles creates a distinct cuffing effect. Created in https://BioRender.com.

Despite its prevalence, affecting nearly 10% of women (Al-Ghadban et al., 2019a), lipedema remains one of the most underdiagnosed and misunderstood connective tissue disorders, often being mistaken for obesity and lymphedema. Lipedema is characterized by surplus accumulation of symmetrical adipose tissue in subcutaneous depots, typically in the buttocks, hips, and thighs, while sparing the feet (Figure 1). In approximately 80% of cases, the upper extremities are also involved, with the tissue accumulation terminating abruptly at the wrists to create a cuffing effect (Al-Ghadban et al., 2019a).

While often mischaracterized as a rare condition, lipedema selectively impacts a significant yet specific segment of the global population. It almost exclusively affects women, and often is initiated during puberty, pregnancy, or menopause, suggesting a hormonal influence (Katzer et al., 2021). 60% of cases also exhibit familial inheritance, suggesting a genetic influence (Poojari et al., 2022). The Lipedema Foundation analyzed the experiences of 521 people with lipedema and found that the age demographic was evenly split across individuals from 30 to 67 years old (Foundation, 2022). The study also found that 89.60% of the surveyed population was white. The Foundation also conducted a technical review of research (as of May 2023) on lipedema and found that the studies reported similar demographics: the majority of those with lipedema were white women between the ages of 35.6–57 years old (Eakin, 2023). It should be noted that race is inconsistently reported in lipedema literature–it is unclear if this is because lipedema is more prevalent in Caucasian people or if lipedema is under-recognized in other races (Eakin, 2023).

Clinicians have reported that lipedema progresses through 3 stages (Table 1), characterized by an increasing accumulation of adipose tissue, and can manifest in 5 different types, classified by where the adipose tissue accumulates (Table 2). These classifications are broadly accepted within the clinical and research community, with some papers defining a fourth stage of lipedema as lipolymphedema, which is the development of secondary lymphedema due to the severity of lipedema (van la Parra et al., 2023; Luta et al., 2025).

**TABLE 1:** Lipedema stage descriptions. Patients may progress through the stages of lipedema or remain at a single disease stage.

Lipedema stage	Lipedema stage description (Herbst et al., 2021; van la Parra et al., 2023; Luta et al., 2025)
I	Skin has a smooth texture. There is loose connective tissue fibrosis, causing the skin to feel pebble-like
II	Skin dimpling appears, and nodules are palpable and more numerous
III	Lipedema tissue is more fibrotic. There are numerous large nodules that can be palpated. Tissue may overhang as it grows in size

**TABLE 2:** Lipedema stage and type descriptions. Patients may have multiple types of lipedema (i.e., in their legs and arms).

Lipedema type	Lipedema type description (Herbst et al., 2021)
I	Lipedema present under the naval, and in the glutes and hips
II	Lipedema present from naval to knees
II	Lipedema present from naval to ankles
IV	Lipedema present in the arms
V	Lipedema present only in the lower legs

Currently, diagnosis occurs through clinical evaluation of the patient (Kruppa et al., 2020). This includes a physical examination and an evaluation of patient symptoms and family history. Diagnostic tests are typically run to rule out other symptoms and dysfunctions, such as abnormal blood pressure or insulin resistance. However, there are no laboratory tests that give an official diagnosis of lipedema (Poojari et al., 2022). Due to the lack of standardized tests and the heterogeneity of its clinical presentation, patients are frequently misdiagnosed, typically with obesity based on overall BMI measurements, or lymphedema due to the localized adipose deposition in the lower extremities (where lymphatics can be blocked). This confusion is compounded by the fact that advanced adipose accumulation can eventually induce secondary obesity and lymphatic dysfunction.

There is currently no cure for lipedema. Conservative therapies for patients include lymphatic drainage, compression therapy, exercise therapy, and dietary counseling (Amato and Benitti, 2021; Herbst et al., 2021). These therapies are typically performed regularly, with the primary goal of preventing secondary complications in patients. The therapies, however, do not cure lipedema and only bring about a small reduction in tissue volume, as it has been observed that lipedema tissue resists changes in diet and exercise (Mazarei et al., 2025). The most invasive treatment for patients is liposuction to remove fat tissue; however, this is only a temporary solution, as the adipose tissue returns, and pain can persist (Mazarei et al., 2025). Clinicians have also researched whether GLP-1 agonists can help reduce lipedema symptoms. Patton et al. studied the effects of exenatide on 5 Italian women with lipedema (of varying lipedema stage and type), and found that 3–6 months of treatment, with or without lifestyle changes, reduced pain symptoms in all patients and led to weight loss in some patients (Patton et al., 2025). Further research into the effects of GLP-1 agonist treatment across larger and more diverse patient populations is needed to understand the mechanisms by which it reduces pain and symptoms for patients.

Physical and mental stressors significantly compromise the health and quality of life of patients with lipedema. They endure a daily burden of pain, fatigue, and swelling, as well as gait abnormalities and leg joint issues (Priglinger et al., 2017; Torre et al., 2018). While they are biologically distinct, the progression of lipedema creates a physiological and psychological environment that significantly increases the risk of secondary obesity (Priglinger et al., 2017; Al-Ghadban et al., 2019a). This is driven by the patient’s reduced mobility, systemic inflammation, and chronic stress-induced cortisol elevation. Furthermore, the weight-loss resistance of the pathological tissue can foster psychological distress, frequently manifesting as eating disorders. Many patients endure body shame, social isolation, and depression, among other forms of psychosocial distress, due to the negative stigma associated with increased body size (Al-Ghadban et al., 2019a; Dahlberg et al., 2025).

While there is still much to be learned in this space, lipedema research has advanced rapidly in recent years. Researchers have focused on characterizing the disease across multiple scales at the clinical, tissue, and cellular levels. The field has begun to identify unique features of lipedema and is focused on investigating the mechanisms underlying its etiology and progression. This review paper discusses current research into the underlying mechanisms of lipedema and emerging *in vitro* models used to study the condition. Finally, this article will highlight recent advancements in adipose tissue *in vitro* modelling that could guide future work in lipedema research, leading to earlier diagnoses and improved patient outcomes.

## 2 Mechanisms of lipedema disease progression

**FIGURE 2:**
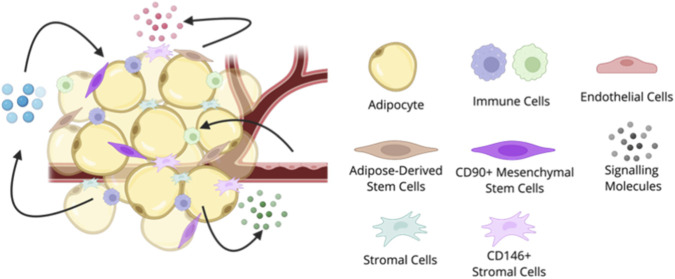
Cellular Constituents of Adipose Tissue. Schematic illustrating the heterogeneous cellular landscape of adipose tissue, comprising mature unilocular adipocytes and the stromal vascular fraction (SVF). The SVF is characterized by a diverse population of adipose-derived stem cells (ASCs), endothelial cells, pericytes, stromal cells (fibroblasts), and resident immune cells. In lipedema specifically, it has been observed that there are higher concentrations of CD90^+^ and CD146+ cells (Priglinger et al., 2017). Created in https://BioRender.com.

**FIGURE 3:**
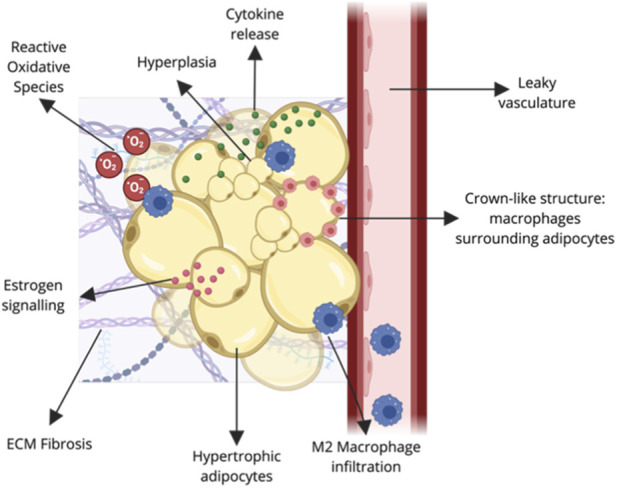
Pathological Alterations in Lipedema Adipose Tissue. Schematic depicting the complex microenvironment of lipedema tissue. Characterized by adipocyte hypertrophy and hyperplasia, the tissue exhibits significant fibrosis and increased vascular permeability. Molecular drivers include dysregulated estrogen signaling, M2 macrophage infiltration, and increased cytokine and reactive oxygen species (ROS) release. Crown-like structures are also present through the tissue. Created in https://BioRender.com.

Lipedema is a chronic disorder of the loose connective tissue characterized by pathological expansion of subcutaneous adipose tissue. Adipose tissue is a complex endocrine organ that is responsible for energy balance in the body, which is regulated through lipogenesis, lipolysis, and hormone secretion. Many cell types make up adipose tissue, including mature adipocytes, immune cells, endothelial cells, adipose-derived stem cells (ASCs)/preadipocytes, and stromal cells (Figure 2). The cell-cell and cell-tissue interactions influence and regulate energy balance and metabolism; where imbalances and changes in the cells and their interactions lead to adipose-tissue disorders, such as obesity and diabetes. It is currently unknown what changes in adipose tissue cause or contribute to the development and progression of lipedema. The mechanisms theorized to contribute to lipedema pathophysiology (Figure 3) are discussed below.

### 2.1 Hormones

Lipedema’s onset typically occurs during puberty, pregnancy, or menopause, suggesting that lipedema is a hormone-related disease and more likely an estrogen-related disease (Katzer et al., 2021). Adipose tissue is an endocrine organ and a site of estrogen synthesis via the aromatase enzyme (Kuryłowicz, 2023). Adipose tissue distribution patterns fluctuate throughout a woman’s lifespan, driven by shifting estrogen levels. Typically, pre-menopause, adipose tissue is distributed in the subcutaneous and femoral-gluteal regions. Post-menopause, decreased systemic estrogen levels direct adipose tissue deposition to the abdominal and visceral compartments (Hetemäki et al., 2021). Estrogen has also been shown to play a role in regulating lipolysis and lipogenesis through its estrogen receptors, ERα and ERβ (Kuryłowicz, 2023). ERα is a positive regulator of GLUT-4, VEGF, and PPAR-*γ* (Katzer et al., 2021). PPAR-γ plays a dominant role in adipose tissue accumulation, as the master regulator of adipogenesis. GLUT-4 is responsible for glucose transport into adipocytes in response to insulin stimulation. VEGF is responsible for initiating angiogenesis within adipose tissue, which is necessary for adipose tissue growth and maintenance. The positive regulation of all three markers leads to adipose tissue growth. Studies on the relationship between ERα and Lipoprotein lipase (LPL) have been contradictory: while some have shown that LPL activity is not affected by estrogen signaling, others have shown that estrogen signaling decreases LPL activity specifically in gluteal adipose tissue (Katzer et al., 2021). ERβ is known to inhibit PPAR-*γ* activity (Katzer et al., 2021). In lipedema, the ratio of these receptors may be skewed, favoring ERα over Erβ. This imbalance could increase GLUT-4, VEGF, and PPAR-*γ* activity, leading to the overgrowth of adipose tissue seen in lipedema. Potential Erα-mediated modulation of LPL could also alter adipose tissue distribution throughout the body, explaining the lipedema phenotype. There may also be a contribution of adipocyte-produced steroidogenic enzymes leading to increased estrogen release. However, further research will need to explore how estrogen-mediated dysregulation occurs.

### 2.2 Adipocytes and ECM

Analysis of lipedema tissue has demonstrated that both adipocyte biology and ECM composition differ significantly from healthy adipose tissue. However, it remains unclear whether these pathological differences trigger the onset of lipedema or are secondary to the disease. Histological assessments have established that adipocyte size is significantly increased, identifying cellular hypertrophy as a hallmark of lipedema tissue (Suga et al., 2009; Al-Ghadban et al., 2019b; Wolf et al., 2021; Kruppa et al., 2023). This comparison was made to non-affected tissue within the same patient (Suga et al., 2009) or to BMI- and age-matched healthy controls without a lipedema diagnosis (Al-Ghadban et al., 2019b; Wolf et al., 2021; Kruppa et al., 2023). The latter 3 studies analyzed patients diagnosed with stages 1–3 of lipedema, with Kruppa et al. specifically observing that adipocyte hypertrophy increased with lipedema stage, particularly within thigh tissue (Kruppa et al., 2023). Furthermore, gene expression analysis of adipocytes differentiated from lipedema-derived stem cells (derived from Stages 1–3 of lipedema) revealed a significant upregulation of adipogenic markers, such as leptin and PPAR-γ, compared to healthy non-lipedema adipocytes suggesting an increased adipogenic differentiation potential in lipedema tissue (Al-Ghadban et al., 2020a). Enhanced adipogenic differentiation may lead to an increased number of mature adipocytes, suggesting adipocyte hyperplasia in lipedema adipose tissue as well.

**FIGURE 4:**
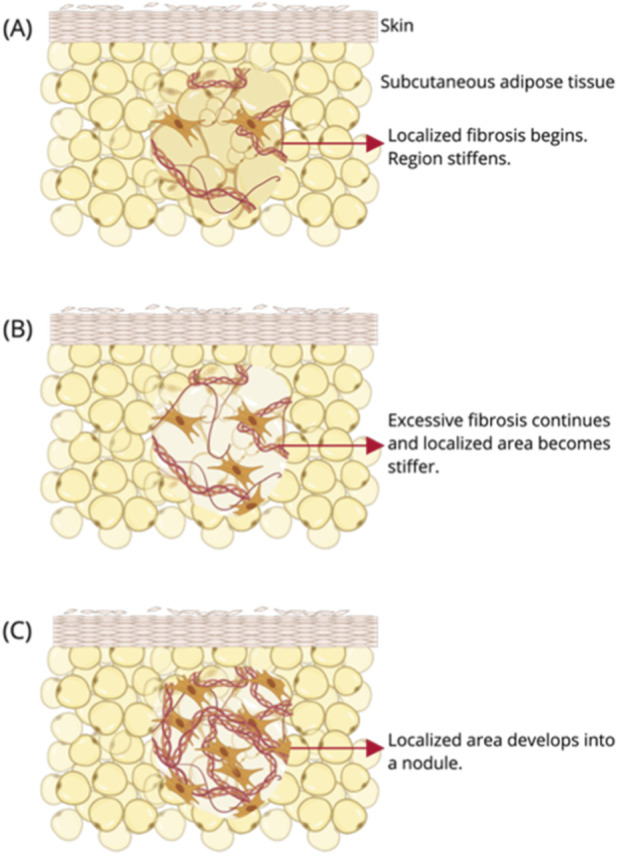
Composition and Presentation of Lipedema Nodules. **(A–C)** depicts the growth of nodules, from the start of fibrosis to the development of a nodule. Lipedema nodules are stiff, fibrotic, hardened adipose masses randomly distributed throughout the tissue. These structures exhibit greater mechanical rigidity than adjacent fat. On a macroscopic scale, the high density of fibrotic tissue gives these nodules a pale or white appearance, distinguishing them from the surrounding yellow adipose tissue. Created in https://BioRender.com.

Similar to obesity, researchers have observed increased collagen content in lipedema tissue, indicating that fibrosis is also a characteristic of lipedema (Felmerer et al., 2020a; Kruppa et al., 2023). This excessive collagen deposition contributes to a stiffer interstitial environment, which may further exacerbate lymphatic dysfunction by increasing tissue pressure. Altered extracellular matrix deposition also results in one unique feature of lipedema, clinically identified as nodules. Nodules are hardened masses that can be felt right below the skin of patients with lipedema (Herbst et al., 2021). The nodules are arranged in no discernible pattern, and as lipedema progresses, grow in size from the size of rice-grains to walnuts (Herbst et al., 2021; Mazarei et al., 2025). The nodules have been clinically described to be hardened fat and heavy with fibrotic tissue (Herbst et al., 2021). Current hypotheses suggest that these nodules represent focal points of excessive ECM remodeling, where collagen and adipocyte clusters become encapsulated (Figure 4). Despite their clinical significance in diagnosis, the specific cues that trigger this localized transition from fibrosis to nodularity remain a critical gap in the current literature. Further research is needed to understand whether this transition is driven by biochemical cues associated with localized hypoxia within the fibrotic regions, mechanical cues arising from local tissue stiffening, or a combination of both. Defining the transition cues for nodule development will be critical in developing therapeutics for growth prevention and pain management.

### 2.3 M2 macrophages and other immune cell involvement

Crown-like structures have been observed in lipedema tissue (Suga et al., 2009; Priglinger et al., 2017; Al-Ghadban et al., 2019b), similar to those observed in obese tissue (Cinti et al., 2005). Crown-like structures are clusters of macrophages surrounding dead or dying adipocytes and serve as markers of inflammation in adipose tissue. These structures were present in all stages of lipedema and were noted through CD68^+^ staining (Kruppa et al., 2023). Kruppa et al. also observed that CD68^+^, CD86^+^ M1, and CD206+ M2 macrophages all increased in population with increasing lipedema stage (Kruppa et al., 2023). The increase of CD86^+^ M1 macrophages indicates an increase in inflammation as the disease progresses (Kruppa et al., 2023).

It has been specifically observed that lipedema is associated with a significant increase in M2 macrophage infiltration in lipedema tissue (Felmerer et al., 2020b; Wolf et al., 2022; Kruppa et al., 2023). M2 macrophages are anti-inflammatory and crucial for wound healing, tissue repair, and ECM remodeling. This observation is in contrast to what is typically observed in obesity, where there is an increase in M1 macrophages (Castoldi et al., 2016), and in lymphedema, where there is an increase in T cells (Duhon et al., 2022). The specific drivers and pathological impacts of M2 macrophage infiltration in lipedema remain poorly understood. While these macrophages are known to secrete TGF-β, which promotes collagen expression and fibrosis, it remains to be established whether this pathway is active in lipedema pathogenesis (Thomas and Apovian, 2017). It is unclear whether the enrichment of M2 macrophages represents an attempt to resolve existing lipedema-associated inflammation (in line with their typical role of healing) or whether the macrophages are pro-fibrotic and promoting dysfunctional repair mechanisms leading to inflammation. Wolf et al. tested the effects of CD163+, a surface-marker that is expressed on M2 macrophages, (through lipedema-conditioned media) on adipose-derived stem cells. They found that after 7 days of differentiation in the conditioned medium, lipid staining increased significantly compared with that of cells differentiated in conditioned medium from healthy control patients (Wolf et al., 2022). While this indicates that M2 macrophages play a role in adipogenesis, further research is needed to isolate the effects of M2 macrophages specifically on adipose tissue and to explore their potential as a biomarker for lipedema.

In contrast to the above studies, Straub et al., observed a suppression of inflammation and immune cell activation after performing transcriptomic analysis of abdominal and thigh subcutaneous adipose tissue of 14 lipedema patients ranging from stages 1–3 (Straub et al., 2025). The authors suggest that the immune cell infiltration may be a result of co-occurring morbidities, such as lymphedema, and that immune cells do not play a causative role in lipedema. Methodological differences are likely to drive the contrasting findings between this study and the aforementioned studies. Straub et al. analyzed all samples together, regardless of adipose tissue location, whereas the above studies performed histological staining and gene expression of specific macrophage markers on thigh adipose tissue biopsies. Differences in sampling location, biopsy depth, patient lipedema stage, and co-morbidities could have also influenced patterns of gene expression. Additionally, all studies were cross-sectional, analyzing biopsies obtained at a single point in time; differences in the stage of disease progression at time of collection could influence the contrasting findings of immune cell involvement.

Other immune cells, such as T-cells and mast cells, have not been reported to be significantly different in lipedema tissue compared to healthy adipose tissue (Al-Ghadban et al., 2019b; Felmerer et al., 2020b). When evaluating inflammatory cytokines, it has been reported that the mRNA expression of IL-6, VEGF, TNF, and IL-1β trended upwards in adipocytes differentiated from lipedema ASCs isolated from the thigh (Al-Ghadban et al., 2020a; Al-Ghadban et al., 2020b). Wolf et al. also found that concentrations of IL11, IL28A, and IL29 were significantly increased in the serum of patients with lipedema (Wolf et al., 2021). More research is needed to evaluate the inflammatory profile of lipedema and to understand how inflammation contributes to its progression.

### 2.4 Oxidative stress

The impact of oxidative stress on lipedema has been explored. Nankam et al. analyzed the plasma of 13 women diagnosed with lipedema at stages 1–3 (lipedema type was not documented). The researchers found significantly elevated levels of superoxide dismutase (SOD), catalase, and malondialdehyde (MDA) (Nankam et al., 2022). SOD and catalase are the first lines of defense against oxidative species, and MDA is a product of lipid peroxidation. Elevated levels of these three compounds suggest that oxidative stress plays a role in lipedema. Straub et al., analyzed oxidative stress levels in 72 lipedema patients (diagnosed with stages 1–3) compared to 49 controls. They found that there were reduced glutamic acid levels (which is necessary for glutathione synthesis) in the serum of lipedema patients, and that there was a trend of reduced glutamic acid/glutathione ratios, indicating increased oxidative stress (Straub et al., 2025). Further research is needed to determine whether oxidative stress is a driver of lipedema or a consequence of the expanding, hypoxic adipose tissue. Understanding the timing of this biochemical shift could also be key to developing targeted antioxidant therapies for patients.

### 2.5 Changes in the vascular system (both blood and lymphatics)

It is theorized that vascular dysfunction leads to excessive interstitial fluid accumulation in lipedema tissue, promoting adipose tissue expansion (Poojari et al., 2022). Current research focuses on the bidirectional relationship between the vascular and lymphatic systems to understand how these vessels may contribute to and be affected by lipedema.

Histological observations suggest that the blood vasculature in lipedema tissue may be morphologically distinct from healthy controls, characterized by higher vessel density, as well as significantly dilated vessels (Al-Ghadban et al., 2019b; Allen et al., 2020). These structural abnormalities, which point toward disordered angiogenesis and increased microvascular permeability, are corroborated by *in vitro* evidence; specifically, lipedema-conditioned media triggers pro-angiogenetic behavior in HUVECS and endothelial barrier dysfunction in primary human endothelial cells (Strohmeier et al., 2022; Al-Ghadban et al., 2023). These findings indicate that the lipedema microenvironment secretes specific paracrine factors that actively drive vascular impairment. Despite these trends, characterizations of the vascular landscape remain divergent across studies. While Al-Ghadban et al. noted increased blood vessel numbers and larger capillary diameters (Al-Ghadban et al., 2019b), and Allen et al. identified a significant increase in the percent total leaky vessels (Allen et al., 2020), Felmerer et al. observed no morphological vascular changes in lipedema patients compared to healthy controls (Felmerer et al., 2020b). Such discrepancies could arise from patients’ disease stage at time of sampling. Al-Ghadban et al., analyzed patients at earlier stages of lipedema, Felmerer et al., analyzed patients at later stages of lipedema, and Allen et al., analyzed patients at all stages. Patient BMIs also trended higher in Felmerer et al.’s and Allen et al.’s study. Furthermore, Allen et al., used different criteria to define an abnormal vessel phenotype. Discrepancies could also arise from patient history, biopsy depth, anatomical type, and co-morbidities. Careful documentation of these discrepancies is important to note for future studies to understand how changes in the vascular system change throughout lipedema progression.

The role of the lymphatic system is also critical in lipedema pathogenesis. Crescenzi et al., found elevated sodium levels in the skin and subcutaneous adipose tissue of lipedema patients’ legs compared to healthy controls (Crescenzi et al., 2018). The group hypothesized that lymphatic dysfunction led to this increased sodium accumulation in the skin and adipose tissue, due to a lack of lymphatic clearance of fluid. This accumulation likely further contributes to blood vasculature dysfunction, as it has been shown that chronically elevated sodium levels can lead to endothelial glycocalyx dysfunction and inflammation (Schierke et al., 2017). As the endothelial barrier fails, the vessels will continue to leak, creating a cycle of fluid extravasation, impaired drainage, and progressive tissue inflammation.

### 2.6 Changes in the stromal vascular fraction (SVF)

To evaluate cellular differences in the vascular microenvironment of lipedema patients, researchers have characterized the stromal vascular fraction (SVF), which encompasses ASCs, endothelial cells, pericytes, fibroblasts, and immune cells. Priglinger et al. demonstrated that the SVF harvested from lipedema patients yielded a higher cell count compared to healthy tissue (Priglinger et al., 2017). Flow cytometric analysis revealed a significant enrichment of CD90^+^ (mesenchymal stem cell marker) and CD146+ (pericyte marker) populations in lipedema SVF, likely accounting for the increased overall cell yield observed in these patients. Building on this cellular characterization, Strohmier et al. used Fluorescence-Activated Cell Sorting to isolate specific subpopulations, including endothelial cells, pericytes, and ASCs from the lipedema SVF (Strohmeier et al., 2022). Subsequent qPCR analysis across these cell types, the total SVF, and whole adipose tissue identified several key dysregulated pathways. Notably, CYP19A1 (encoding aromatase enzyme responsible for androgen to estrogen conversion) was significantly upregulated in both lipedema adipose tissue and the SVF. Furthermore, ZNF423, an early master regulator for adipogenesis, was significantly upregulated in lipedema-derived endothelial cells and pericytes. At the tissue level, ITGAX (CD11c), was significantly elevated, suggesting an altered immune profile. By integrating analysis of the SVF with that of the whole adipose tissue, these studies provide more insight into how lipedema manifests across multiple cell lineages and how their interplay perpetuates the disease phenotype.

## 3 *In vitro* lipedema modelling

Some of the mechanisms discussed above have been studied using *in vitro* models that attempt to recapitulate the *in vivo* adipose tissue environment to elucidate cause-and-effect relationships that are not possible in patients. As there are currently no animal models of lipedema, *in vitro* models are crucial. Unlike endpoint analyses in lipedema patients, which rely on tissue biopsies, blood samples, and clinical observations (such as nodules) that occur after the disease has already manifested, *in vitro* models allow for longitudinal study of disease initiation. These model systems can test hypotheses with highly defined boundary conditions, assessing multiple biochemical or mechanical stimuli in parallel.

Many 2D monolayer cultures have provided foundational data essential for hypothesis building in the field. However, 2D models fail to capture the complex 3D architecture of lipedema tissue. 3D models allow researchers to manipulate the microenvironment’s stiffness, thereby mimicking the fibrotic transition and/or nodule formation observed in lipedema. Furthermore, by integrating patient-derived cells into these environments, *in vitro* models can serve as tools to evaluate confounding variables, for example, decoupling patient predisposition and extracellular matrix cues.

### 3.1 Cell type considerations for *in vitro* models

In adipose tissue engineering, research has traditionally focused on ASC culture. Historically, the murine-derived 3T3-L1 preadipocyte line has been the most common model for studying aging, obesity, and adipogenesis (Zoico et al., 2010; Etesami et al., 2020; Crosthwait et al., 2025). Cell lines offer the advantages of immortality and high reproducibility, facilitating long-term, continuous experimentation without needing to maintain low passage numbers. However, their primary drawback is a lack of physiological relevance to human tissue due to interspecies differences and the genetic alterations that occur during immortalization. To address these limitations, the use of primary human ASCs isolated directly from patients has gained traction. While this approach, already utilized in lipedema research, offers superior biological relevance, hASCs are limited by finite availability and phenotypic drift during extended passage (González-Cruz and Darling, 2013). Consequently, induced pluripotent stem cells (iPSCs) have emerged as a promising alternative, providing a virtually unlimited, patient-specific cell source. Robust protocols now exist to differentiate human iPSCs into various adipocyte lineages, including white, brown, and beige, allowing for the study of insulin resistance, inflammatory profiles, and metabolic disorders (Aghadi et al., 2022; Friesen et al., 2022; Qi et al., 2022; Qi et al., 2023; Rao et al., 2023). Characterizing lipedema through this lens will be vital for understanding how the disease alters progenitor function and differentiation.

Moving beyond monocultures, co-culture models have been developed to explore the crosstalk between ASCs and other lineages, such as myocytes and immune cells (Nitta and Orlando, 2013; Yadav et al., 2020; Kuppusamy et al., 2021). These systems better reflect the heterogeneity of *in vivo* adipose tissue and are essential for analyzing the paracrine signaling driving the disease phenotype. However, 2D co-cultures often consist of only 2 cell types due to spatial constraints and limitations in nutrient diffusion, thereby failing to capture the full cellular complexity of the adipose microenvironment.

A further challenge in modeling is the culture of mature adipocytes, as their high lipid content makes them buoyant and causes them to float in standard 2D culture. While the “ceiling culture” technique was developed to circumvent this, it often induces a morphological shift from unilocular to multilocular phenotypes and presents a risk of dedifferentiation of the mature adipocytes as well (Sugihara et al., 1986; Sugihara et al., 1987; Côté et al., 2019; He et al., 2022; Kim et al., 2022). These changes in the mature adipocytes’ defining characteristics compromise the model’s physiological relevance and prevent the “ceiling culture” technique from accurately replicating lipedema pathology over long-term culture. These limitations underscore the need to transition to more advanced 3D platforms that can stabilize mature adipocytes and support multicellular architecture, as will be discussed later.

### 3.2 2D models of lipedema

Many 2D *in vitro* models of lipedema have utilized adipose-derived stem cells (ASCs) isolated from patient samples (Al-Ghadban et al., 2020a; Ishaq et al., 2022; Strohmeier et al., 2022; Wolf et al., 2022; Al-Ghadban et al., 2023; Al-Ghadban et al., 2024). Al-Ghadban et al. ran a full phenotypic characterization of ASCs harvested from the thighs of lipedema patients, comparing them to both intra-patient abdominal ASCs and those from healthy controls. They found that lipedema ASCs have greater adipogenic differentiation potential than their healthy counterparts. This was further supported by an increase in leptin and PPAR-*γ* expression in lipedema-differentiated adipocytes compared to healthy-differentiated adipocytes (Al-Ghadban et al., 2020a). Complementing these phenotypic findings, Ishaq et al. ran a comparative multi-omics analysis of ASCs from lipedema and non-lipedema patients (Ishaq et al., 2022). qPCR and western blotting revealed a significant upregulation of Bub1 (a mitotic checkpoint gene) in lipedema ASCs. Further analysis indicated that Bub1 upregulation promotes ASCs hyperproliferation in lipedema. Using ASCs to study lipedema provides insight into potential biomarkers, such as Bub1, that could be used to diagnose lipedema and serve as a therapeutic target to arrest pathological tissue growth.

Since lipedema involves significant vascular changes, researchers have increasingly incorporated endothelial cells into their disease models. Specifically, there has been a focus on paracrine signaling by incorporating conditioned media to evaluate the impact of secreted proteins on endothelial behavior (Strohmeier et al., 2022; Al-Ghadban et al., 2023). Al-Ghadban et al. investigated the effects of conditioned media from both lipedema-derived ASCs and differentiated adipocytes on 2D HUVEC monolayers. Compared to healthy controls, they observed an upregulation in expression of angiogenic, inflammatory, and ECM remodeling gene markers (specifically MMP9, leptin, and HGF) (Al-Ghadban et al., 2023). Using a different cell source, Strohmeier et al. applied lipedema-SVF-conditioned media on human endothelial cells to assess changes in endothelial junction morphology and functionality (Strohmeier et al., 2022). The group noted alterations in CD31 and ZO-1 localization at endothelial junctions upon exposure to lipedema-SVF conditioned media. Subsequent permeability assays and qPCR revealed a significant increase in endothelial permeability, accompanied by a significant downregulation of CDH5, a critical adherens junction protein. Interestingly, when screening the conditioned media for protein secretion levels against healthy controls, only a significant decrease in IL-8 was detected (Strohmeier et al., 2022). Using conditioned media, both groups demonstrated that cellular crosstalk may drive vascular dysfunction in lipedema.

Current 2D models have proven invaluable for characterizing the unique properties of lipedema-derived ASCs and the SVF, as well as the fundamental paracrine interactions between ASCs and other cell types in adipose tissue. Thus far, these models have provided critical insights into the lipedema disease phenotype and have identified promising biomarkers for both diagnostic and therapeutic applications. While 2D models remain essential for high-throughput, cost-effective mechanistic studies, they are limited by their inability to replicate the ECM, mechanical stiffness, and 3D spatial cues that govern cell-cell interactions and disease progression. Consequently, the field also requires 3D models to more accurately recapitulate the native lipedema microenvironment.

### 3.3 3D models of lipedema

To overcome the limitations of 2D cultures, researchers have developed 3D cell culture models, such as spheroids, that better recapitulate the *in vivo* microenvironment and enable controlled experimental perturbations. Al-Ghadban et al. utilized lipedema-derived ASC spheroids to evaluate adipogenesis, ECM remodeling, and the effects of estrogen, compared to healthy controls (Al-Ghadban et al., 2020b; Al-Ghadban et al., 2024). While differentiated lipedema spheroids exhibited significant upregulation of adipogenic markers, including PPARG, LPL, and SLC2A4 (Glut4), these levels were similar to the trends observed in healthy controls. However, lipedema spheroids uniquely displayed elevated IL6 expression, and adipocyte-differentiated spheroids had a significant downregulation of MMP2, MMP9, and MMP11.

In another study, Al-Ghadban et al. treated their spheroids with 17β-estradiol, which is the most potent and naturally occurring human estrogen (Al-Ghadban et al., 2024). They demonstrated that the hormonal response is heavily dependent on the model’s dimensionality. In a 2D monolayer platform, estrogen enhanced proliferation and a mesenchymal marker (CD73) in hormone-depleted healthy versus lipedema ASCs. ERβ expression was significantly higher in lipedema ASCs and spheroids, while ERα and GPER levels were markedly reduced in estrogen-treated lipedema spheroids. In healthy cells, estrogen primarily stimulated the expression of CYP19A1 and LIPE; while in lipedema-differentiated cells and spheroids, it significantly upregulated PPAR-γ2 and ERα. This divergent hormonal response, in which the 3D architecture reveals estrogen sensitivities that are entirely absent or reversed in 2D, suggests that the spatial organization and ECM interactions of lipedema tissue are fundamental to its pathophysiology. These findings underscore the need to investigate hormonal fluctuations in a 3D context to identify the biological triggers of lipedema. Understanding these receptor shifts provides a specific target for future research: determining if local estrogen metabolism within the 3D niche can be modulated to arrest tissue growth.

## 4 Future directions - utilizing 3D adipose tissue technologies for lipedema research

As the lipedema field moves toward more comprehensive models, several 3D technologies from general adipose research offer high potential for lipedema applications. These include scaffold-based, scaffold-free, and organ-on-a-chip models.

To more accurately replicate the native ECM, researchers have increasingly utilized scaffold-based models composed of natural or synthetic biomaterials (Halbleib et al., 2003; Cheung et al., 2014; Kaplan et al., 2025). As the primary structural component of the adipose ECM, collagen is a widely used substrate for culturing ASCs and adipocytes (Louis et al., 2017; Louis et al., 2019; Park et al., 2020; Zhang et al., 2025). For example, 3D collagen nanofibers seeded with human-derived ASCs demonstrated cytocompatibility and supported robust adipogenic differentiation (Zhang et al., 2025). Collagen scaffolds also provide the advantage of being stiffness-tunable, providing a platform to isolate and study the effects of ECM-stimulated mechanical cues on adipose tissue and fibrosis (Di Caprio and Bellas, 2020). This would be especially beneficial for lipedema research, as a means to study the causes of fibrosis and nodule development. Furthermore, composite scaffolds, such as collagen combined with alginate or gelatin, improve nutrient diffusion and mechanical flexibility. These hybridized models have successfully supported ASC viability and facilitated co-cultures with macrophages, creating *in vitro* insulin-resistant platforms for screening anti-obesity and anti-diabetic therapeutics (Park et al., 2020). However, a notable limitation of collagen is its inherent bioactivity; because it provides potent cell-specific signals, it can sometimes interfere with specific cellular responses being studied *in vitro*. To overcome this challenge, non-native natural polymers, such as silk fibroin, have been used. Silk scaffolds have been instrumental in developing adipose models that incorporate all the relevant adipose tissue cell types, including mature adipocytes, ASCs, stromal cells, and endothelial cells (Abbott et al., 2016). Silk-based platforms seeded with primary adipose tissue obtained from surgical procedures have maintained cellular viability and metabolic function for up to 3 months. These durable systems allow for longitudinal study of disease triggers, serving as a versatile platform for investigating diverse adipose-related disorders (Abbott et al., 2016; Wang et al., 2017). Applying these scaffold-based technologies to lipedema research would enable long-term studies of mature adipocytes and other critical cell types within a biomimetic, patient-specific environment.

In contrast to scaffold-based approaches, researchers have begun to employ scaffold-free 3D models that leverage cells’ innate capacity for natural self-assembly and endogenous ECM production. These models, which range from single-cell type spheroids to more complex, multi-lineage organoids, aim to recapitulate the native tissue architecture without the influence of exogenous materials (Taylor et al., 2020; Mandl et al., 2022; Dariolli et al., 2025). For example, Taylor et al., who developed an SVF-based adipose tissue organoid that integrates ASCs with critical immune components, including macrophages and mast cells (Taylor et al., 2020). Given that chronic inflammation is a hallmark of both obesity and lipedema, incorporating these immune populations provides a good representation of the *in vivo* microenvironment. The platform demonstrated the ability to track lipid changes during adipose differentiation and showed significant lipidomic shifts following stimulation with inflammatory mediators such as LPS and IL-4 (Taylor et al., 2020). Various methodologies facilitate the development of these organoid models, including the hanging-drop technique, magnetic bioprinting, and orbital stirring for self-aggregation (Muller et al., 2019; Mandl et al., 2022; Dariolli et al., 2025). By eliminating the risk of scaffold-induced signaling interference, scaffold-free organoids provide a physiological environment for studying the cell-cell interactions and paracrine signaling that drive disease conditions. Implementing these multicellular organoid models in lipedema research will enable high-fidelity analysis of how progenitor and immune cells collectively contribute to the pathological tissue architecture.

To replicate the physiological fluid dynamics of native tissue, organ-on-a-chip models have become an important tool in adipose research. By introducing microfluidic flow, these platforms recapitulate the dynamic *in vivo* environment, mimicking nutrient delivery and hemodynamic shear forces that govern cell viability, metabolic function, and the vasculature’s phenotype. Given that lipedema is characterized by significant microvascular impairment, incorporating a functional vascular component is essential for developing high-fidelity *in vitro* models. An important development in this area is the fully autologous, human, immunocompetent white adipose tissue-on-a-chip (WAT-on-a-chip) developed by Rogal et al. (2022) . This “mix-and-match” system allows for the integration of various SVF subpopulations with mature adipocytes and endothelial cells. The platform maintained cellular viability for over 12 days, with mature adipocytes preserving their characteristic unilocular morphology and dense spatial arrangement. Furthermore, the model established an endothelial barrier with identifiable tight junctions, remaining functional throughout the culture period. Further functional assays confirmed adipocytes maintained their energy storage capacity and mobilization, while inflammatory challenges with TNF and LPS successfully induced significant cytokine upregulation (Rogal et al., 2022). While many adipose tissue-on-a-chip platforms have been used to study obesity and metabolic disorders (Yang et al., 2021; Compera et al., 2022; Leung et al., 2022; Huff et al., 2024) their application to lipedema remains a frontier for the field. Adopting this technology will enable researchers to investigate how pathological shear flow and endothelial permeability contribute to the progression of the condition, offering a sophisticated environment to assess vasculature-targeted therapeutics and drug efficacy.

## 5 Conclusion

Lipedema is a prevalent but disproportionately understudied and underdiagnosed disorder. Despite an expansion in research efforts, significant gaps in knowledge remain regarding its etiology and pathological progression. The current lack of definitive diagnostic tools and targeted therapies leaves patients to navigate chronic pain, frequent misdiagnosis, and psychological burdens.

Current research has focused on characterizing the lipedema microenvironment. Through various *in vitro* platforms, researchers have identified key features of the disease, including adipocyte hypertrophy, fibrosis, chronic inflammation, and vascular dysfunction. These models have enabled the study of aberrant adipogenesis, paracrine signaling, and hormonal responses to determine how lipedema-derived adipose tissue is functionally impaired. Both 2D and 3D platforms offer distinct advantages, and responses may differ across models. 2D models offer a cost-effective, high-throughput means of mechanistic screening, while 3D models offer the physiological relevance, spatial architecture, and mechanical cues necessary to recapitulate the altered tissue microenvironment of lipedema.

The tissue engineering field has developed sophisticated technologies, including iPSCs, scaffold-based and organoid 3D cultures, and organ-on-a-chip systems, that hold immense potential for lipedema research. Implementing these technologies in the field is critical, as the ability to model complex tissue-level interactions under long-term, experimentally controlled conditions will drastically enhance our understanding of the disease’s triggers. Furthermore, these technologies offer the advantage of being patient-specific, providing a powerful tool for drug efficacy screening and personalized therapeutic development.

As clinical and academic recognition of lipedema increases, so too will the demand for improved patient care and early interventions. Developing advanced, high-fidelity models is essential to unraveling the disease’s underlying mechanisms, ultimately informing better diagnostic and therapeutic strategies to improve patient outcomes.
